# Understanding the Meaningful Places for Aging-in-Place: A Human-Centric Approach toward Inter-Domain Design Criteria Consideration in Taiwan

**DOI:** 10.3390/ijerph20021373

**Published:** 2023-01-12

**Authors:** Tzen-Ying Ling, Hsien-Tsung Lu, Yen-Pin Kao, Szu-Cheng Chien, Hung-Chou Chen, Li-Fong Lin

**Affiliations:** 1 Department of Architecture, Tamkang University, New Taipei 25137, Taiwan; 2 Department of Orthopedics, Taipei Medical University Hospital, Taipei 11031, Taiwan; 3 Department of Orthopedics, School of Medicine, College of Medicine, Taipei Medical University, Taipei 11031, Taiwan; 4 Graduate Institute of Biomedical Informatics, College of Medical Science and Technology, Taipei Medical University, Taipei 11031, Taiwan; 5 Engineering Cluster, Singapore Institute of Technology, 10 Dover Drive, Singapore 138683, Singapore; 6 Department of Physical Medicine and Rehabilitation, Shuang Ho Hospital, Taipei Medical University, New Taipei 23561, Taiwan; 7 Department of Physical Medicine and Rehabilitation, School of Medicine, College of Medicine, Taipei Medical University, Taipei 11031, Taiwan; 8 School of Gerontology and Long-Term Care, College of Nursing, Taipei Medical University, Taipei 11031, Taiwan

**Keywords:** human-centric attributes, place-of-aging, aging-in place, domain interpretation gap

## Abstract

Background: Aging is key to inclusion, and it should be taken into account when designing every place of human activity. However, the implementation of such guidelines often fails the human-centric aspiration as health and design domain interpretation gaps impede the suitable reading and implementation strategies. Purpose: This study aimed to understand critical factors in the place-of-aging and to examine the gap in domain interpretation affecting age-friendly housing. Methods: Using grounded theory as a base, questionnaire interviews were implemented either face-to-face or through an online process by health and design domain experts. Overall, 40 respondents (20 health and 20 design experts) evaluated the key criteria to prioritize according to their value of importance. The factor analysis resulted in the stated deviation, suggesting a necessity to redefine the attributes of the dwelling based on a people, place and process framework. Results: The systemic analysis affirmed the inter-disciplinary gap to enhancing the dwelling provision. The health domain experts consistently ranked the criteria higher or equal than the design domain except for safety and security criteria. Both domains agreed that affordability is a main concern, as elders must be able to afford their dwelling choice. Conclusion: The valuable finding of the key criteria in the study is to uphold the value of the urban health resilience implication as the core of this study.

## 1. Introduction

Cities have large numbers of older citizens and are home to 43.2% of the older population [1]. The apparent spatial unevenness and place-embedded implications of population aging nowadays suggests a significance need to adjust the scale-driven and spatially-based attributes for aging-in-place [2]. Places in which people grow old are hostile and challenging, presenting potential barriers to the implementation or design consideration of ideal aging-in-place [3]. This requires a cohesive understanding and linkage of people and environment to align within health resilience thinking. The physical, social, and service aspects of communities and housing options should be enhanced [4]. To this end, possible measures require preplanning, which is critical and necessary. The aging population presents numerous challenges to the built habitat coping with providing adequate services to the inhabitants; that includes the design of the living environments [5]. Given the needs of an aging society, the planning and design of cities, communities, and housing should be centered on age-friendliness to strengthen a community’s functions and create innovative housing products [6]. It is ascertained that ‘age-friendly’ housing should be suitable for residents of any age; the location or place, provision of multigenerational multi-unit living arrangements, and suitable spatial arrangement should be the focus [7]. With the new demographic structure change, it means that the housing conditions, economic security, health care, and comfortable living environments for older adults should be carefully examined [8]. In addition, it is ascertained that long-term care and the improvement to the prevailing dwelling condition for the aging are worth further research.

Although acknowledging that the place-of-aging is important, intervention-based medical pharmacological and high-tech perspectives are customarily the finite solutions being considered by the health domain nowadays. The built environment—that is, human-made surroundings—are rarely considered but worth exploring [9]. For health-related professions, space is regarded as locations within a Cartesian world that, in and of themselves, have no meaning [10]. To age-in-place, the focus should be on improving the dwelling experience: that is, physical and non-physical understanding gathered [11,12]. The demand creates an impetuous attention to understand the state of being for the aging demographic; this is crucial in creating effective and acceptable dwelling and care that is tailored to their unique needs. Of course, the dwelling should provide adequate living spaces for as many healthy years as possible [13]. The person-centered spatial accommodation can improve the quality of life in older adults. Yet, this definite interpretation gap among the health and design domain has brought forth an implementation barrier to a collaborative effort for adequate place-of-aging. Certainly, a broader integrated approach in allowing spatial complexities in the formulation of an implementable solution could facilitate more effective aging-in-place [3,9,11]. Not much is known yet about how to translate these preferences into practical living concepts and architectural design [14]. An optimized living concept should categorically consider the architecturally feasible combinations of dwelling while considering various aging-friendly urban attributes highly recommended by the WHO [15,16]. The systems fail at the weakest link—the built environment [9]. Even if the living quarters are equipped with universal accessibility devices, the place could still be ill-fitted for aging people with or without impairments or frailty of any kind [17,18,19,20,21]. Disparate guidelines require cohesive planning from the health and design domain, hence the purpose of this study. 

The optimization of the opportunities for health, participation, and security to enhance quality of life as people age is important [22]. Aging is key to inclusion, and thus, it should be taken into account when designing every place of human activity [9]. Environmental gerontology acknowledges the distinctive link between the aging process in conjunction to the physical environment [23]; tools have been developed to address environmental needs for older adults [21,24]. However, the implementation of such guidelines often fails the human-centric aspiration as health and design domain interpretation gaps impede the suitable reading and implementation strategies. To foster urban health resilience, an inter-disciplinary consensus could realize more productive results [13]. This study aims to determine the divergence in the health and design experts’ opinion as well as a suitable criteria framework model for assessment application. The place-based familiarity and environment are key drivers of living preferences. The social and habitat domain of functional ability is pertinent to the thematic priorities of aging friendly and urban places [25].

## 2. Materials and Methods

### 2.1. Study Site

Taiwan became an aging society in 1993, and subsequently, an aged society in April 2018 when its aging older population grew to 14.10% of its total population [26]. Taiwan has witnessed a rapid demographic aging at a rate that is more than twice that of European countries and the United States [27]. Currently, one in five people is an older adult [28], meaning above the age of 65. The sub-replacement fertility rate has ameliorated the declining birth rate. Taiwan recorded only 181,601 births in 2018 and a low birthrate of 165,249 births in 2020 [29]. In a relatively short period of time, Taiwan needs to respond swiftly to accommodating the aging demographic. To encourage the promotion of places for the aging, the Ministry of Health and Welfare’s Health Promotion Administration (HPA) initiated the Age-friendly City Awards to encourage government agencies to draft public policies that include elderly health and build a supportive environment for senior citizens. There are 1043 elderly welfare institutions, including 42 nursing homes and 1001 long-term care institutions: the demand for elderly housing far outweighs the current supply. Policies such as the” Senior Citizens Welfare Act” promulgated in 1980 created incentives to set up new apartments and elderly communities.

**Figure 1:**
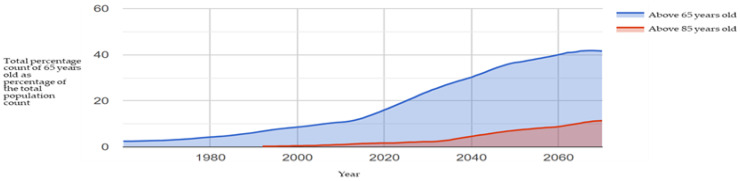
Percentage of those 65 years old to the total population in Taiwan; statistics from MOI 2022 and prepared by this study.

The local Taiwanese society retained a traditional culture based in Confucianism. Notably, this is based on respect for the aged and wise. At the same time, the family structure’s change in Taiwan has shifted dramatically in recent decades. Aside from the urban nuclear family dwelling, the rise in living with spouse only, living alone, living with relatives and friends or in elderly care and nursing institutions means a lack of cohesive planning to accommodate the aging population. The state lingers as the birthrate continues to decline and the aging demographic continues to rise (see Figure 1).

**Figure 2:**
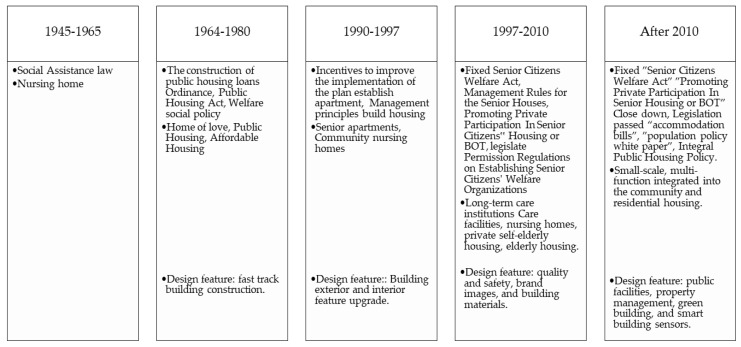
Development of dwelling feature thinking in Taiwan. Source: Lin, 2012, CEPD 2010.

With Taiwan becoming an ‘aging’ society in 2018, the island-state continued its rapid aging process coupled with a declining birth rate. Aside from the urban nuclear family dwelling, the rise in living with spouse only, living alone, living with relatives and friends or in elderly care and nursing institutions means a lack of cohesive planning to accommodate the aging population. In a relatively short period of time. The Social and Family Affairs Administration under the Ministry of Health and Welfare reported a total of 1084 nursing homes and retirement communities in Taiwan collectively looking after 51,000 older residents in Taiwan [30]. At the same time, the family structure’s change in Taiwan has shifted dramatically in recent decades (Figure 2).

In the 1990s, private sectors began to invest in nursing homes constructions: Pacific took over the Peitou “Sesame Hotel “ and converted it into Taiwan’s first senior housing, the Chang Gung Health and Culture Village in 1992; the Ruentex Group foresaw a future society of platinum opportunities, and they started planning and construction in 1993 [27]. As a needs-oriented approach, user-specific user needs are assessed, but the implementation phase should take into account aging societies and sub-replacement fertility rates. In other words, age friendliness is central when planning such housing options. This phase focuses on two concepts: that is, emphasizing the notion of lifetime homes, which entails accessible design, universal design, and open building, so as to meet the spatial adjustment needs caused by changes in age. 

Although the long-term care plan 2.0 introduced by the MOHW in 2017 began to promote the concept of aging in place, the 10-year holistic undertaking seeks to embed care services in the community as well as secure long-term care providers. Aging-in-place, however, requires a retrofit of the interior space and infrastructure; the improvement should be renewable or customizable according to one’s needs. The current home layout based on the nuclear family need must transform it into a multigenerational aging-in-place (in the home). Elders have adopted this concept as a preferred choice and a prevailing trend for seniors in Taiwan [17,31].

‘Age-friendly’ housing is still at large in many built communities. The demand has surged; 33.83% of the total population aged between 55 and 65 are willing to stay in nursing homes agencies, apartments for older adults and community shelters that allows aging adults to look after themselves in their future life. However, only 19.46% of adults aged over 65 accept this living style, and the highest reluctance is from the group aged 65 to 69. To that end, improving access to health care is essential to advance the quality of life; the private sector in Taiwan has begun to advertise aging-in-place as a viable option for prospective buyers by increasing amenities and convenience within the development. However, divergence from the implementation strategy in retrofit or a new design for age-friendly housing creates a confusing state; the design and health domain continue to examine the criteria in disparate and divergent manners.

### 2.2. The Place-of-Aging: A Perspectival Understanding

Aging inheres accumulating a biological handicap that affects the physical and mental capacity. From the physical health perspective, conditions include poor vision, arthritis, urinary incontinence, hearing loss, reduced reflexes, poor balance and mobility problems [32,33]. Aging-in-place views aging as a normal part of human growth. It allows aging at home and aging in a familiar habitat. The aging process should allow for a natural evolvement for older adults in the settings they live in. The place-of-aging then is an integrated concept that consists of incorporating the idea of aging-in-place and allowing the flexibility of multigenerational living or care arrangements within a lifetime home. The WHO (2019) expanded the critical consideration to include the role fulfilled by environments that are supportive for more older adults in numerous areas (health, long-term care, transportation, housing, work, social protection, information, and communication). Public spaces around the place-of-aging should be accessible and allowing mixed-use activities.

To stimulate and support urban aging, an initiative of the World Health Organization instilled the program for cities to meet the needs of older citizens. Thus, allocating a proper physical environment, civic participation, fostering place for social interaction, and community support must come into place [3]. To consider where the aging residential population moves, location choices come to mind. It shows that senior households move to places with highly valued consumer amenities including health care, high temperatures and low taxes [34], which are often closer to their place of birth. Elders prefer to age-in-place; not only does the familiarity reinforce a sense of attachment to home and neighborhood, it improves well-being and social connectedness [32,35,36]. Affected by the built environment, elders’ mobility is also a crucial concern for aging-in-place [5]. 

Policies to stimulate the older adults to live independently at home for as long as possible have been promulgated by various governmental agencies [33]. It permits residents to stay at home and participate in the community in a safe manner [37]; they can retain their independence, familiarity and comfort regardless of age, income, or ability level [38]. Eight domains within the living context are recommended by the WHO: affordability, essential services, design, modifications, maintenance, aging-in-place, community integration, and housing option [39]. Mostly detailed as a public health guideline, these recommendations have not translated into a spatial design framework. Moreover, although there is a preference to age in the community as “aging in place”, healthcare and design experts in the essential constituent to provide service and the spatial environment have not agreed; to realize a cohesive understanding, all stakeholders must strive to create a common ground regarding a sense of well-being relating to a person’s goals, functional capacities, and opportunities [40]. Universal accessibility aids are not a panacea for the psychosocial integration of older adults [41,42].

It is imperative that both perception and physical needs be addressed especially for people with multi-morbidities who require solutions of increased complexity and variety [9]. The aging population are forced to dwell in ill-functioned homes or in communal nursing homes. Professional stakeholders should consider such multi-faceted criteria [23,43,44]. As the health domain continues to set new guidelines, the design domain rarely places user experience in terms of ordinary built environment as a subject matter during the architectural design process [9]. For example, in Western housing spaces, environmental barriers for older adults, especially for those with functional limitations, are common obstacles observed in many homes [45]. Similar cases exist in Taiwan, as the design domain follows a mainstream Western architecture education and training. As the new health-related guidelines are being set, the obstacle immensely enlarges the hard-to-reach interpretation challenge for the design domain. Therefore, discussion based on lifetime neighborhoods, livable communities and age-friendly cities necessitates an inter-disciplinary strategy to advise suitable spatial action plans [3,33,46,47].

Active aging indicates a process of optimizing opportunities for health, participation, and security in order to enhance quality of life as people age […], allowing people to “[…] realize their potential for physical, social and mental well-being throughout the life course” [48]. Conversely, physical inactivity could accelerate the aging process. In this regard, a proper living setting in a familiar setting could increase the social and physical engagement, thus contributing to the well-being state for aging-in-place.

### 2.3. Human-Centric Criteria for Aging-in-Place

Proper age-friendly environments help foster healthy aging in two ways: by supporting the building and maintenance of intrinsic capacity across the life course and by enabling greater functional ability so that people with varying levels of capacity can perform the things they value [48]. Proper social contact in the community provides support for access to resources and can prevent isolation and loneliness [49]. Between physiology, perception and the habitat, the gap remains at large. Gaps in the provision of adequate habitat for assisted housing and home environments prompt a need for proactive measure to resolve the manifold standards for practice or regulatory structures for the design domain [34].

To comprehend the multidimensional nature of active aging, the input from the health perspective can contribute to the understanding of the individual micro (person), meso (process), and macro systems (place and policymaking) based on health (prime) environments [50]. Taking a resilience stand imposes a challenging yet propitious notion implementable in community planning; it solicits a neighborhood to acquire a “malleable” state, which is attainable via a flexible adaptive process inclusive of diverse disciplines and stakeholders [13,51] to satisfy populations with greater longevity; the result is a friendlier demographic aging [52]. The spatial practice regards that the ‘domesticity’ must not only refer to local typologies but also resident’s subjectivity, movement, physicality, identity, and functions served [13].

**Figure 3:**
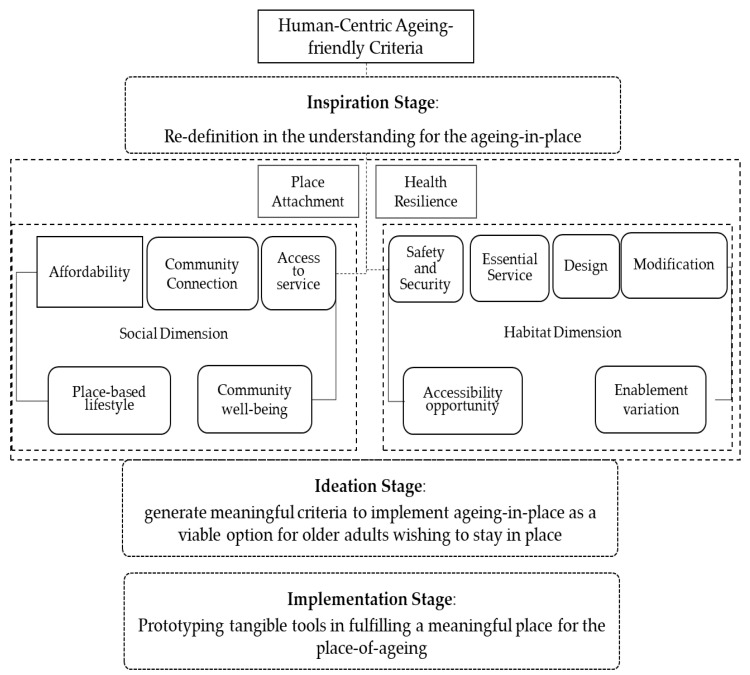
Revised human-centric criteria for aging-in-place. Source: modified from WHO housing-friendly checklist.

This study ascertains the multi-criteria from the current WHO framework, examining the social and habitat-related factors involved (Figure 3). The main intent should focus on taking the preferences and values of the individual and his or her relatives into account when deciding on the provision of care, which has major implications on the quality of life in older adults [46,53]. The process in human-centered consideration iteratively unites innovative thinking through inspiration, ideation, and finally an implementation phase [54,55]. The focus produces a dual-centric realm to the research. This study argues that through the inspiration phase, a redefinition in the understanding of the aging-in-place should contest the known assumptions in the existing framework and begin to define the critical issues pertinent to the older adult’s facilitation to age in place [56]. Professional stakeholders participate in the ideation to generate meaningful criteria to implement aging-in-place as a viable option for older adults wishing to stay in place. Finally, the attained process could generate an implementation framework to further test and refine assumptions, prototyping tangible tools in fulfilling a meaningful place for the place-of-aging. Place attachment and health resilience trigger an adaptive process in which the transitioning for aging-in-place may happen. Due to lack of exposure or grasp on how the built environment affects human perception and physiology, the design domain remains unaware of the impact that space may have on the mind and body of the aging population.

Figure 3 identifies the dimensional consideration in aging-friendly housing to aging-in-place. Human-centric evaluation places attention on such processes that should encourage prolonged engagement and new insights on how the life experiences of older adult’s shape preferences, beliefs, and habits [13]. In terms of the consideration process, we proposed the processional consideration as follows:

1. Inspiration Phase: The social and habitat dimensional criteria are considered. Attribute criteria characterized by the ability to afford and facilitate daily life in the community; the ability to conduct day-to-day activities in place, diversity in keeping habitual lifestyle, and connectivity with social network are pertinent to aging-in-place. Within this consideration, the affordability, community connection, access to service, place-based lifestyle and community well-being are part of the inclusive evaluation to attain a social satisfaction toward aging-in place.
2. Ideation phase: As per the re-defined criteria, the time of restructuring of the dimensional criteria is obtained. Within the social–spatial habitat domain, the focus is placed on attaining meaningful place attachment as well as optimized health resilience. A high correlation of place, people, and process should be weighted.
3. Implementation phase: As per the restructured thinking, the dimensional criteria could be edited into the human-centric focused framework within the social–spatial factors. This is characterized by a domain-friendly and realizable design that reduced anti-social spaces and low interaction among the residents within the community. Newly defined criteria are derived from the restructuring of decisive factors and characterized by the domain inter-mixed issues and accrued understanding from weighted analysis.

### 2.4. Assessment Method

Utilizing grounded theory as a method of inquiry and a resultant product of that inquiry [57], the aging-in place issue references in the WHO’s friendly aging recommendation as a base, we utilized the human-centric design thinking to regard and separate the criteria by the people (social) and place (habitat) factors [3,58].

This study aims to identify ‘concerned variables’ based on the realistic preferences, values, attitudes, behaviors, and decision-making process among the health and design domain knowledge. Twenty experts from the health domain, including gerontologists, nurses, and medical doctors, were invited. From the design domain, architects, interior designers, and contractors were asked to respond to the questionnaire. Key questions are further assessed through the descriptive and exploratory multivariate analysis. 

Taking the participant observation stand, the questionnaire interview process allows local domain groups to assess the issues and concerns faced by experts. The participating experts expressed their opinions as an active participant and an objective observer in facilitating the aging residents in their place-of-aging. The assessment process was conducted either through face-to-face or through an online questionnaire; the responses were measured by the domain expertise of the respondents’ assessment in the scale of importance in regard to the spatial or social impacts on the aging population and their dwelling condition. Perceptions of surroundings differed from individuals depending on domain and experiences attained from domain expertise; from these, subjective and objective criteria were judged and a scale of importance was given. These critical differences could not be evaluated using other means. The cumulative result from the interactions and experience gathered by the domain experts were measured as the derivative to the direct spatial and/or social dimensions; in turn, it measured the criteria concerning aging residents’ aging-in-place in responding to related measures and impacts involved. 

**Figure 4:**
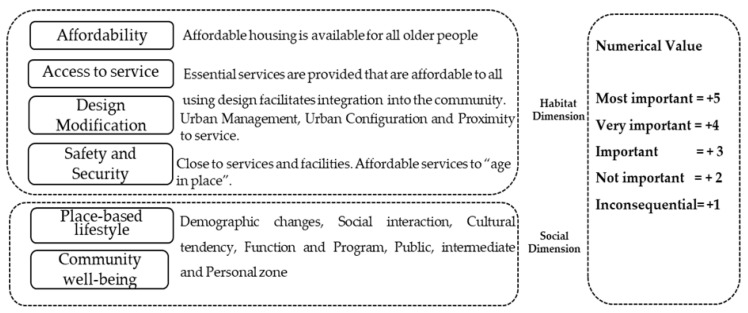
Dimensional criteria and assessment value.

The assessment criteria framework is as follows: In the inspiration phase, a domain consideration questionnaire was given to professional stakeholders (Figure 4). Then, the quantitative evaluation based on the questionnaire was conducted. The numerical quantifier of attributes relevant from the two expert groups is collected; there were a multitude of defined concerns during the design stages, as outlined by the WHO AFFC, from which critical differences were focused, which allows for the amplest scope for revealing divergent motivations incentives. The experts were invited to return a questionnaire between December 2020 and January 2021; the study utilized mutually anonymous evaluation of the questionnaire to prevent undue influence by certain members of the team [59]. The quantitative assessment is divided into eight domains to describe the framework; the comparative factors were then further analyzed to obtain an improved understanding of the housing features that enable aging-in-place.

To assess the critical factors in the ideation phase, this study ascertains that both the physical and social realms are key determinants for consideration to remain healthy, independent, and autonomous long into their old age. Within the housing checklist provided by the World Health Organization [39], this study proposes to integrate the attributes into two main categories of social and habitat consideration. Factor analysis as a statistical method is used in this study to allocate variability among observed and correlated variables [60]. affirmed that 20 to 50 variables are suitable but emphasize that fewer variables can be used. Furthermore, [61] notes that for the study data to be considered suitable for factor analysis, the correlation matrix should show at least some correlations of r = 0.3 or greater. Finally, in the implementation phase, this study proposes a revised consideration framework, taking into account the quantitative result for critical factors. The human-centric thinking could obtain a more meaningful process in the aging-in-place for older adults while in keeping the valuable place attachment and health resilience.

## 3. Results

### 3.1. Respondents’ Demographic Characteristics

Questionnaire interviews were implemented either face-to-face or through an online process. The combined group of 40 respondents (20 health and 20 design experts) in Taiwan evaluated the key criteria to prioritize according to their value of importance. The health domain experts comprise doctors, gerontologists and nurses engaging in elderly care. The design experts practice the design of buildings and interiors; most of them have experience in the design of buildings or interior retrofit for elderly adults. We underscore the eight attributes as proposed by the WHO, asking each domain expert to evaluate the importance of each attribute’s criteria in the implementation for aging-in-place. The eight attributes were given on their ranking importance by both domain experts. If respondents felt that the place-of-aging currently lacks features that were thought to be important for a place for aging, the questionnaire also asked respondents to elaborate on attributes that necessitate focus or attention.

Prospective criteria consideration in terms of social well-being and adaptability were illustrated. In total, 40 respondents were selected to represent age, gender, domain-specific experiences, and part-taking opportunity in the implementation of aging-in-place in the local communities in Taiwan. Most health domain respondents were clinical nurses, gerontologists, and medical doctors. The design domain comprises architects, interior designers, contractors or architecture education-related professionals. Respondents must have practiced within the profession for at least 5 years. Respondents’ ages varied, with the youngest being in their late twenties and the eldest in their 60s. The results reflect the feedback from the respondents. 

### 3.2. Inspiration Phase

#### 3.2.1. Respondents’ Results by Inter-Domain Comparative Assessment

**Figure 5:**
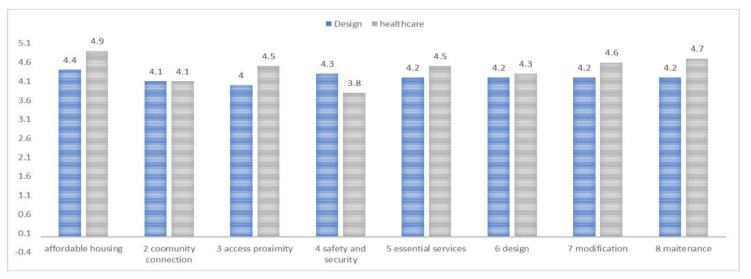
Comparative assessment result from health and design domain experts.

Both domain groups evaluated each criterion for the result of the weighted importance, specificity, and assessment toward the criteria to fulfill aging-in-place. From Figure 5 below, the consideration is the ranking per domain on the eight criteria evaluated by health and design domain experts. The respondents’ weighted assessments are illustrated in Figure 5.

In the overall comparative weighted assessment, health domain experts consistently ranked the criteria higher or equal than those in the design domain except for safety and security criterion. This is due to the legal responsibility of the design domain to ensure user’s safety in the physical habitat. Both domains agreed that affordability is a main concern, as elders must be able to afford their dwelling choice. It is therefore a shared interest that could be deemed a public issue. For community and connection criterion, both domains ranked equally, reflecting the importance of connecting to the community and having adequate interaction with others. Community connection criterion was ranked equally by both domains, as aging-in-place requires the health domain’s directive guidance as part of the overall health consideration. This guidance directive has spilled over to the physical space requirement that must be implemented by the design domain. However, it is ascertained that most of the criteria rely on the design domain to be implemented adequately in the physical habitat. The guidance implemented by the health domain could be assistive or disruptive, as the knowledge gap and difference in the criteria-weighted importance is clearly reflected through the comparative assessment result. By domain, the design domain respondents evaluated the affordability as being the most important, followed by safety and security, with essential service, design, modification and maintenance ranked equally with a numerical value of 4.2. Access and proximity ranked lowest with a value of 4; this may be justified, as access to other amenities or space beyond the legal boundary is not the design expert’s legal responsibility through design intervention. The health domain ranked safety and security lowest at 3.8, which was followed by community connection at 4.1 and design at 4.3; these were most probable because it is beyond the health domain’s legal or domain expertise and dependent on the elder’s ability to connect socially or with another domain’s practice; it is therefore independent of the health domain probable expertise interventions. Affordability and maintenance criteria were ranked highest, at 4.9 and 4.7, which reflect the domain’s understanding and concern over the dwelling habitat condition for the older adults. Overall, we observed a disparity between domains. The health domain remains the active voice directing the guidance on the aging adult care, while aging-in-place requires more intervention from the design domain. This clear gap in domain disparity reflects the state of gerontology in the elder care field as well as a design-incongruent practice to afford the best living condition for the aging population. 

#### 3.2.2. Respondents’ Result by Domain Expertise and Criterion

**Figure 6:**
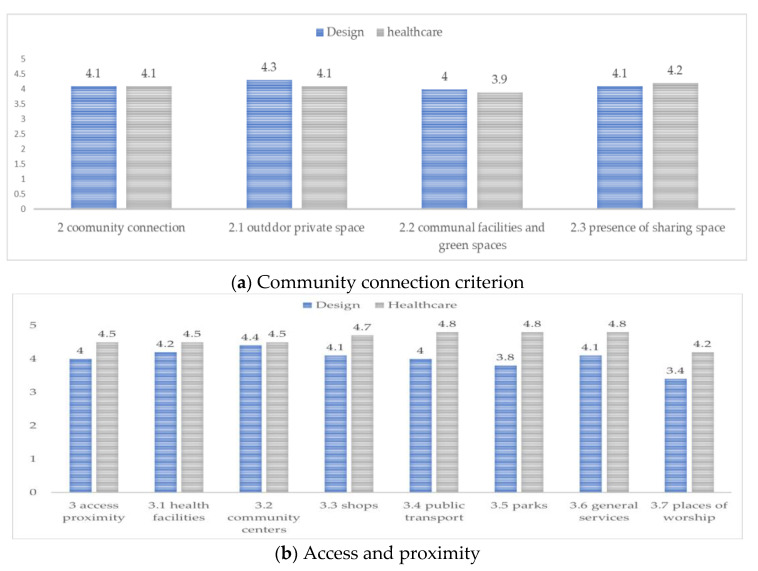

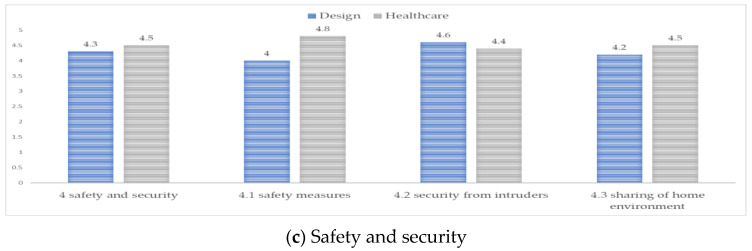
Aging-in-place dwelling criteria: (**a**) community connection; (**b**) access proximity, and (**c**) safety and security.

By each criterion grouping, the average weighted average is shown in Figure 6 below.

**Figure 7:**
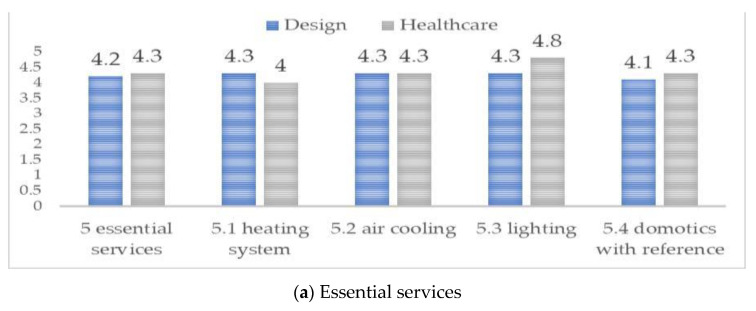

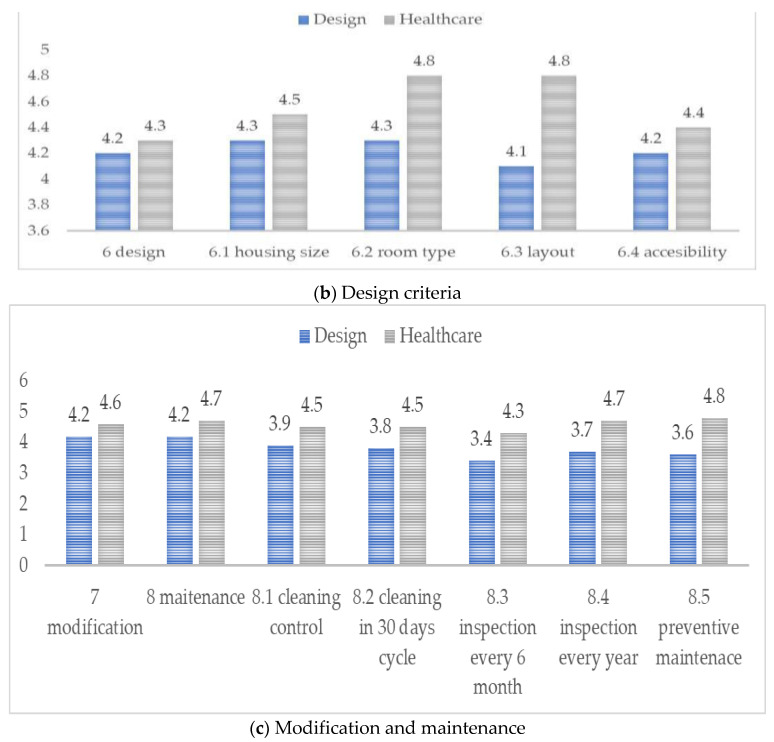
Criteria 5–8 result; (**a**) essential services; (**b**) design criteria, and (**c**) modification and maintenance.

For the community connection criterion shown in Figure 6a, the aim is to stipulate the importance of outdoor spaces and buildings, including the provision of visual and audio cues and adequate crossing times, street lighting and accessible elevators, ramps within the approach to the dwelling and the community public space. Both domains ranked similarly, although differences do exist in perception and importance value. For outdoor private space, the design team ranked a score of 4.3, which was followed by the presence of sharing space and then communal facilities and green spaces. The health team felt otherwise, ranking the presence of sharing space most significant with a score of 4.2, which was followed by outdoor private space then communal facilities. For Figure 7b, access and proximity, design experts scored lower than healthcare experts. Design experts ranked community centers the highest, with a score of 4.4; health experts, however, ranked public transport, parks, and general services as equally important with a score of 4.8. Health experts regard community auxiliary services much more important than the design experts. In Figure 7c, the safety and security criteria are weighted; design experts ranked safety measure and security from intruders equally significant, while health experts ranked safety from intruders first followed by sharing of the home environment.

The divergent views on attributes of essential services, design criteria and modification and maintenance are affirmed (Figure 7). Design experts held equitable balance in the criteria for essential service (Figure 7a). Health experts ranked domotics the highest, which is followed by heating and lighting; the cooling system was ranked the lowest. Interestingly, domotics is building automation for a home, which is often referred to as a smart home or smart house. It monitors and/or controls home attributes such as lighting, climate, entertainment systems, and appliances. For design criteria (Figure 7b), the design experts adhered to an unbiased view on all the criteria alike, while the health experts ranked room type and layout highest by housing size and accessibility. Design experts felt that design attributes must be evaluated holistically; health experts ranked sporadically. We observe the most visible divergence in the design criterion. Those in the health domain evaluated this criterion highly, giving a value of 4.3 to 4.8, especially for room type and layout attributes. This could be seen as a confirmation that the health domain prioritizes the spatial features, which are expressed in the layout and room type configuration. For the modification and maintenance attributes (Figure 7c), health experts predominantly ranked higher than the design experts, which represent a direct divergence of opinion. Design experts view modification as a necessary improvement due to need or age, while the health experts view each criterion as an added element to the physical environment. Overall, the domain expertise difference in views and importance is apparent. As the need for quality aging-in-place continues, there is a need to obtain a consensus and strategy.

#### 3.2.3. Ideation Phase-Comparative Factors Analysis

The divergent views from the health and design experts represent the divergent shift within the socio-physical provision. The dynamic variance involves a divergence in need of integration. The component factor analysis extracted three main factors relevant to the aging-in-place design consideration for dwelling on Table 1 shown below.

The results show that six of the 28 items are highly correlated. The statistical procedure reveals three meaningful clusters of variables. The design-based social linkage has been affirmed. The social attributes constitute the most important variable; the environmental linkage follows as the physical attribute is desirable. This is critical in the provision for the main transport or venue for the aging-in-place. Lastly, the safety-based well-being factor denotes how safety and security in one’s home and community contribute toward the social well-being in an aging person residing in the neighborhood. Two variables in the essential services domain (air conditions and lighting) and one variable in the design domain (housing size) showed high correlation with design-based social linkage in the factor analysis. The air conditions, lighting system and housing size enhanced the indoor comfort, mood, safety, and well-being of more older adults living in Taiwan. Healthy experts agreed that the above essential services assisted with conditions such as depression, dementia, and frailty.

Three variables of maintenance domain (cleaning and control interventions, inspections, checks, revisions, or replacements) showed high correlation with design-based social linkage in the factor analysis. The opinion of health experts was that the maintenance domain was considered a significant task to ensure a safe and a healthy home environment. The predictive maintenance has been assumed as the most effective strategy to adopt the risk assessment. The assessment exploration of health care and design experts allows an understanding as to how to accommodate friendly aging design principles in the housing or dwelling, allowing for resilient practice to be realized Conceivably, the solution is to categorize three leading factors as a plausible alternative to the urban aging-in-place dwelling design that accommodates the increase in aging population or changing demography. Furthermore, it could be more attentive to the changing household age composition and create more resilience-conscious dwelling and urban public accessibility.

#### 3.2.4. Implementation Phase: Adopting People, Place and Process as Main Consideration

**Figure 8:**
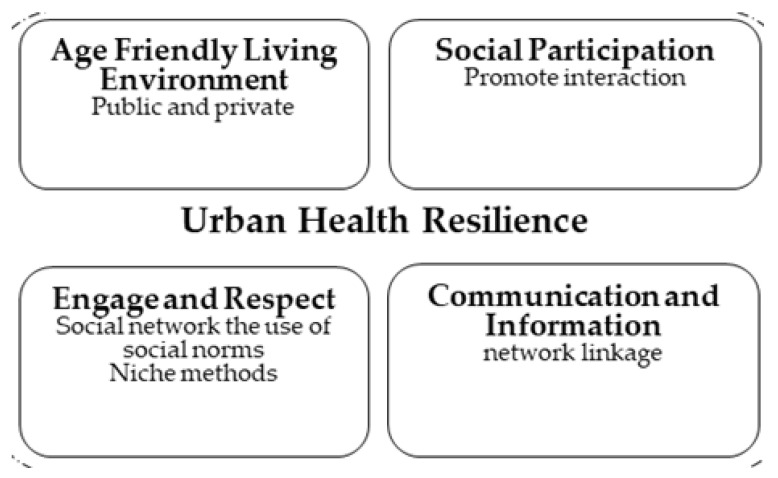
Dimensional principles affecting the urban health resilience and aging-friendly housing.

The social relevance dimension takes people-specific attributes into consideration, while the habitat relevance dimension considers spatial attributes integrated into a comprehensive assessment. The cohesive interlinkage enables health resilience and fosters place attachment. Since the quality and quantity of our social relationships have been linked not only to mental health but also to morbidity and mortality [62], a decisive inter-disciplinary understanding benefits the realization of age-friendly spaces for the elderly adults [63]. AIP (aging-in-place) must include the process of frequent physical, behavioral, as well as social adaptations among older adults, which illustrated the intersectionality of space and time [64]. One essential aspect of delivering human-centered place and process must focus on people’s participation, specifically, collaboration and engagement [65]. A proper environment fulfills a key role in maintaining the functional ability of older adults or facilitating comprehensive attention and health care [48]. In fact, the habitat relevance is crucial in achieving health resilience. Ideally, to promote proper physical activity levels and regular exercise, health-driven initiatives from public agencies in many countries provide guidelines for physical activities for all age groups [66,67] with full details on scientific proven health benefits, which have been published in the United States [US Department of Health and Human Services]. Therefore, the local environment holds its own physical and social identity based on its dominant features and constructed by a collective attribution; each resident uses the self-identification of the city and the built environment with its connotative meanings, which influences a person’s identity. The concept for aging-friendly housing is shown below in Figure 8.

**Figure 9:**
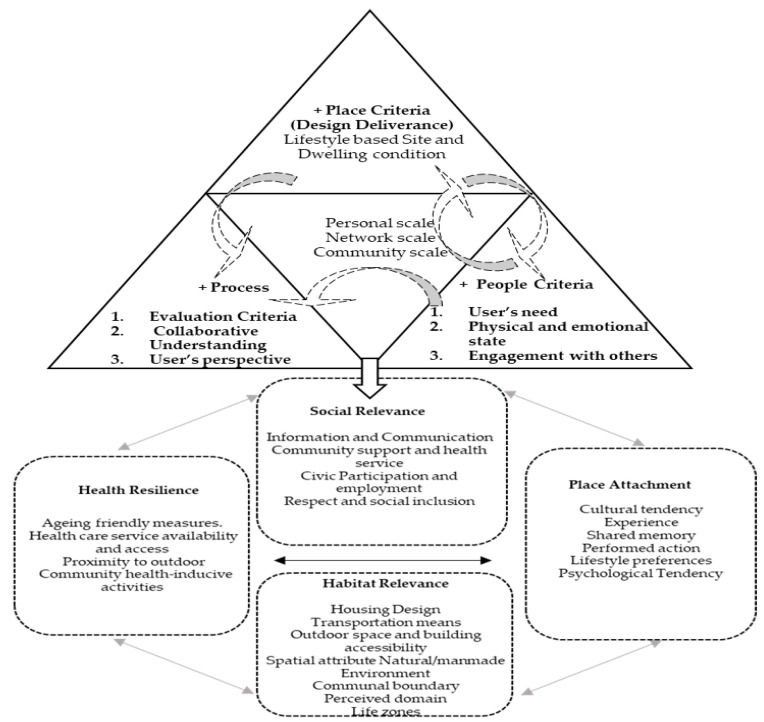
Re-distributed criteria for aging-in-place.

To attain the necessary health resilience, aging-in-place should consider the habitat and the possible interactions among residents in the community. While the health domain has resorted mainly to technology-assisted device and sensors, the design domain still has difficulty in realizing a successful human-centric solution. This result is an entity that has a social dimension and a very real physical basis [68]. Factors such as natural light, landscape features, and design that enables small group clustering should be weighted [69]. Therefore, the proposed structure for the design thinking is as per Figure 9 below.

Design-related practice regarding domestic settings and the building regulations tend to be diverted by place-specific issues related to the interface between human health and interior design [9]. Often, as residents become frail, the aim is to adapt the domestic environment with home care assistance in their own homes [68,69]. However, throughout the process of design decision making, useful design guidelines are necessary to successfully carry out the design [70]. Aside from the health domain consideration, it is argued that communities should provide services and opportunities tailored to the needs of older residents to enable active participation [48]. Social isolation and loneliness could be linked to the way that our environments inhibit meaningful social encounters [71]. The design thinking for the place-of-aging and its physical habitat is seldom considered in the framework proposed by the WHO. The divergence in domain expertise within lays in the difference in understanding between the two domains. While the health domain emphasizes the safety and connective power within the social realm, the design domain focuses on the physical attributes that enhance the activities generated within the daily living routine. Therefore, the affirmation of the design-based social linkage should bring forth a more detailed focus on the contribution of design in the provision of human-centric place-of-aging. The social attributes constitute the most important variable as considered by both domains; the environmental linkage follows as the physical attribute is desirable as the main transport or venue for the aging-in-place. 

Lifestyle represents a crucial element in aging-in-place. As such, factors contributing to healthy living and better sustenance in social engagement should be instilled. A growing body of evidence supports the role of design-led social attributes, as aging-in-place in a familiar setting is conducive to active living. Given the pivotal role of space in health, a “healthy” lifestyle must include the design and provision of appropriate living space. It is a primary non-genetic factor affecting elderly adults’ health and lifespan. A better understanding of the underlying mechanisms is beneficial to designing space conducive for healthy lifestyles and to develop appropriate design interventions aimed at enhancing the living habitat. The human-centric approach focuses on the people, place and process criteria cohesion. The three factors of design-based and people-based social linkage, place-based environmental linkage and process-based social well-being attributes should be incorporated as the core thinking when devising the living space for place-of-aging. To understand the people’s daily routine and activities is quite important; it should be a place-based observation and analysis; further, each place has a unique geo-cultural specificity that requires an indigenous strategy; lastly, the process in which this is achieved is perceptibly distinctive. Obviously, the current universal framework approach should give avenues for the design domain’s interpretation, since the methodology encompasses common design mechanisms that could target human-centric measures for healthy place-of-aging.

## 4. Discussion

### 4.1. The Meaningful Places and Human-Centric Inclusive Thinking

From a well-being perspective, inclusive thinking in the design of housing and the communal amenities for seniors could utilize health-related measures to increase the mobility and help to ease the transition through the process of aging. However, divergence in the interpretation and perception of physical environment could interfere with a satisfactory experience of aging-in-place. People-centric thinking should be encouraged. Lacking such thinking could mark a definite inequality within the social and physical determinant of health for the aging residents and quality of life in seniors. Encouraging elderly adults to conduct physical activity could prevent frailty, reduce the risk of chronic illnesses, improve cognition and reduce anxiety. On the contrary, inactivity and a sedentary lifestyle could decrease one’s health condition. This is a critical factor that should be addressed in the design process but seldom realized. Furthermore, a lack of cohesive and integrated thinking has caused inadequate and inferior solutions as the outcomes of age-friendly dwelling approaches seldom approximate the assumed targeted outcome within the dwelling habitat. To promote better understanding of the experiences of aging-in-place in changing environments around the world, steady cooperation among and between professional health care and spatial design stakeholders’ contribution is essential to provide the adequate living environment.

Increasingly, communities are adopting the concept of lifetime homes. This encapsulates a broader interpretation of the suitability of housing, extending to the whole life course of a person. Designing for the targeted user group has become a core issue for the health and spatial design professionals. While it is clear in the literature that ‘aging-in-place’ is much preferred, a resilient living habitat demands further attention. It is ascertained that developing understanding of the needs and preferences of aging societies will be crucial to assist in the provision of suitable housing and sustainable communities in the long term. This thinking links to universal design, which is aimed at satisfying the needs of all inhabitants, regardless of age or need. However, the large concentration of aging residents in the community visibly demands a design that answers to the demographic change. Developing understanding is crucial to assist in the provision of suitable housing and sustainable communities in the long term. The design criteria are recognized as a key factor in determining their quality of life and health. This reflects users’ needs and preferences; functionality, cognitive inclination and sensory capacities derive from place-based environmental and sociocultural characteristics, environmental interventions such as adaptations and changes to the physical and social domains/aspects. 

A knowledge gap in promoting mobility and health resilience as related to the people–environment is evident; comparatively less academic research is available on older adults’ preferences and related environment attributes. Health resilience is central to an age-friendly city and the Sustainable Development Goal of inclusive, safe, resilient and sustainable communities. Measures such as the MIPAA (Europe) and Age-friendly cities and communities (AFCC), as well as those of local institutions, have addressed the issue of adjusting the habitat to promote active and healthy aging as well as instilling an increase in quality of life and well-being. Therefore, higher-density neighborhoods are needed that support a range of green infrastructure and transport, affordable housing, and vibrant, exciting, sociable, human-scaled pedestrian experiences. 

The importance of person–environment interaction comes to play as the ’interrelatedness of place’ [2]; clearly, the spatial experience linked to human perceived understanding is crucial and absolutely critical to enable a friendly place for aging. The human-centered design approach generates knowledge by taking the users’ need into account and professional stakeholders formulating viable solutions. This action-oriented knowledge for sustainability emerges when working in integrated ways with the many kinds of knowledge involved in the shared concept for the design, enhancement, and realization of a people-centric spatial realm. On one hand, ecological theory of aging highlights the physical contexts and the home environment in promoting or restricting quality of life and healthy aging; however, disparity in the meaning and translation toward the spatial dimension is still in the infancy stage. At an urban scale, an age-friendly city optimizes opportunities for health, participation, and security to enhance quality of life [39]. It should offer a supportive environment that enables residents to grow older actively within their families, neighborhoods, and civil society, and it offers extensive opportunities for their participation.

### 4.2. Urban Resilience: The Cohesion of a Social–Habitat Domains 

Resilience has been widely discussed as an interactive and multifactorial process that involves unique aspects: the environmental context, the quantity and quality of vital events, and the presence of protective factors adhering and ensuring the necessary quality to maintain the living habitat. These conditions are irrevocably linked with sustainability in all its facets—social, economic, cultural, and environmental—and present enormous challenge and opportunity for the design community. Adequate social support demands a robust build-up of interpersonal relationship; the aging process is individual, multidimensional, and multi-determined in nature; it needs to be evaluated in relation to the positive aspects that can contribute to healthy aging or daily living needs. The most pressing challenge for the urban centers is to provide the adequate dwelling for the aging population.

Taiwan has been developing dwellings merely on a basic needs basis. Not only are the more refined features omitted, it is not even considered for specific users such as the older adults or mixed-age community. Based on the result of the factor analysis, three main themes seem to be in accord because of the myriad of inter-disciplinary exploration. Socially, active aging or aging-in-place both assume the place-based social linkage and support. While the community should provide the necessary social network, proper accessibility among members of the community stimulates the beneficial interaction. To illustrate the high levels of resilience, the aim of aging-in- place should be to align with the urban health resilience. To utilize design as a tool to adhere to the social-based spatial traits necessitates better understanding of the needs of the residents within the dwelling place. The aim is to activate and encourage better interaction among the users. 

In an aging society, this type of thinking should be incorporated into the architecture design. From the design domain’s perspective, urban resilience is about holistically looking into what a given community needs; this includes the social, environmental and changes within the habitat such as extreme climate, earthquakes, floods, and disease outbreaks. The holistic and comprehensive approach of planning is integral to preparing for disasters and supporting older adults as they age in place. Indeed, the social–habitat factor is pivotal in the fulfillment of aging-in-place, which is followed by environmental linkage as correlating to social and habitat relevance to acquire the place attachment and well-being.

### 4.3. Aging in Place-Dwelling Criteria

Housing and other accessible environments including the type of accommodation, the location of accommodation, and the availability of necessary supports and care is pivotal in facilitating active aging-in-place. Sociocultural issues and personal well-being are currently the priorities of sustainable development as the human-centered approach, as people, culture, place and the living process 

The research focuses on the social and habitat realm while incorporating the factors that the WHO deemed important to create an aging-friendly environment in an urban place. The social realm deals with social attributes; asides for affordability, the community connection and access to service are important in maintaining the cohesive place-based lifestyle and social well-being. Factors such as how an aging person can easily access and have the greatest mobility is key. The greatest accessibility afforded through design can stimulate social interaction. The habitat factor focuses on the physical environment within the housing place. The physical environment component is characterized by Ronald John [72] as the “impersonal environment”, which is mainly composed of the physical attributes of the neighborhood, or in this case, the place-of-aging.

The living environment and social integration are critically important for achieving the active aging as well; active aging is inseparable from the social interaction with family members, friends, and neighbors. For the built communities needing modification in the dwelling, efficient planning of the necessary retrofits within the building becomes a necessary move. Design experts must familiarize these needs to realize suitable spatial experience. However, to modify the dwelling to allow effective transition to aging-in-place, the dwellers must spend more cost and time as well as inconvenience. Spatially, the phenomenological approach of ‘place attachment’ observes three features in which the place becomes pivotal for aging-in-place, a familiarity of physical or spatial attributes; a strong attachment to the in-place social network; and reaffirmation of self by lifelong experiences in each place. Unambiguously, friendly and accessible spatial traits could still stimulate positively older adults experiencing exclusion and loneliness mismatches between personal needs and environmental options to fulfill these requirements, which can undermine well-being in later life. Furthermore, a failure in providing adequate place-of-aging could bring forth a psychological experience of inequality that has a profound effect on health. As such, aging-in-place is a goal that enables older adults to maintain a suitable lifestyle that is conducive to the independence in the daily life as well as a sense of wellbeing that contributes to a high quality of life in their home or in their community. 

## 5. Conclusions

Considering that our aging society needs adequate dwelling and place for friendly interaction and communication as well for numerous activities to take place, this study ascertains that crucial policies to guide and encourage urban centers to fortify and enhance the spatial and social infrastructure are critical in maintaining the place-based advantage. In an aging society, communities necessitate design thinking to convert their public and personal amenities geared to the aging residents in accord with public institutional criteria. The process is faced with severe spatial-temporal challenges. Presently, the design guidelines necessitate architects/designers to execute and evaluate the strategies set by health care experts at their own discretion. 

Taiwan has become part of the aging community while the infrastructure and service for the aging population continues to evolve. In the process, many new developments claim to incorporate aging-friendly features within the dwelling; they are mostly tech-related devices or sensors as an add-on element. The field of environmental gerontology is still at an early stage, making the inter-disciplinary advancement quite disparate. Most importantly, the spaces that the elders consider part of their daily life must be improved to ensure unobstructed mobility, taking into account adequate functional capacity and the space retrofit process in relation to aging. 

This study ascertained that there is an awareness between both domains that space and place matter as they exemplify the essence of meaningful lifestyle in aging-in-place. There is divergence in opinions among domains; this raises an opportunity to calibrate critical parameters within the social and habitat realm. Spatially, the current practice and guidelines for implementing aging-in-place should look into the process of people and place-based definition. Design experts agreed that most attributes are purely within the design consideration, as human safety is placed at the upmost importance per code and practice ethic. However, design is defined as a singular attribute with similar importance as other attributes. As expected, health-care experts positively re-affirmed the WHO position and evaluated every attribute almost equally important. Faced with such disparity, the aim is to simplify to three leading factors or categories: that of the social and environment linkage as well as safety consideration. The revised assessment could essentially bridge the gap of deviation that each expert group may hold. As the common denominator for the interdisciplinary consensus, design intervention and implementation require the “user-centric” thinking to provide the friendliness factor into the habitat to strengthen the community and civic support. These considerations encourage the place attachment and, consequently, the desired urban resilience needed in the community. The reshuffled structure could be used as an applicable tool. Further study should expand the framework to define a user’s preference and in-place adaptation as parameters in the prototype development within the urban context.

## Figures and Tables

**Table 1:** Factor analysis resulting in three main factors.

Criteria Consideration	Design-Based Social Linkage	Place-Based Environment Linkage	People-Based Social Well-Being
1. Affordability	0.085	**0.217**	0.031
2 Community Connection	0.108	**0.467**	0.072
2.1 Presence of outdoor private space	0.222	**0.328**	0.149
2.2 Overlooking to open or green space	0.117	**0.324**	0.003
2.3 Presence of sharing space	0.050	**0.394**	0.125
3. Access of public amenities	0.013	**0.492**	0.002
3.1 Proximity to health care	**0.385**	0.190	0.002
3.2 Proximity to community center	**0.475**	0.133	0.079
3.3 Proximity to retail shops	0.157	0.159	**0.375**
3.4 Public transport accessibility	**0.203**	0.069	0.010
3.5 Park accessibility	**0.478**	0.042	0.000
3.6 General service accessibility	**0.290**	0.021	0.166
3.7 Place of worship accessibility	0.146	0.007	**0.498**
4 Safety and security measures	**0.198**	0.063	0.055
4.1 Safety measure availability	**0.377**	0.024	0.093
4.2 Security against intruders	**0.545**	0.011	0.042
4.3 Private sharing space at home	**0.508**	0.000	0.011
5 Essential service availability	**0.365**	0.004	0.157
5.1 Heating system availability	**0.714**	0.010	0.000
5.2 Air conditioning/cooking equipment	**0.476**	0.062	0.133
5.3 Lighting availability	**0.263**	0.178	0.107
6 Design service	**0.276**	0.179	0.190
6.1 Housing area size	**0.315**	0.121	0.292
6.2 Room type selection	**0.444**	0.300	0.031
6.3 Layout variation	**0.591**	0.129	0.012
6.4 Accessibility at free will	**0.645**	0.057	0.017
7 Modification flexibility	**0.685**	0.067	0.035
8 Maintenance	**0.465**	0.116	0.100

Note: bolded numbers denotes factors with relevant importance.

## Data Availability

Not applicable.

## References

[1] OECD (2015). OfEC-oaD: Ageing in Cities.

[2] Skinner M.W., Cloutier D., Andrews G.J. (2015). Geographies of ageing: Progress and possibilities after two decades of change. Prog. Hum. Geogr..

[3] Lewis C., Buffel T. (2020). Aging in place and the places of aging: A longitudinal study. J. Aging Stud..

[4] da Silva Júnior E.G., Eulálio M.D.C., Souto R.Q., de Lima Santos K.D.L., de Melo R.L.P., Lacerda A.R. (2019). The capacity for resilience and social support in the urban older. Ciênc. Saúde Colet..

[5] Grazuleviciute-Vileniske I., Seduikyte L., Daugelaite A., Rudokas K. (2020). Links between heritage building, historic urban landscape and sustainable development: Systematic approach. Landsc. Archit. Art.

[6] Forsyth A., Molinsky J., Kan H.Y. (2019). Improving housing and neighborhoods for the vulnerable, older adults, small households, urban design, and planning. Urban Des. Int..

[7] Fulmer T., Patel P., Levy N., Mate K., Berman A., Pelton L., Beard J., Kalache A., Auerbach J. (2020). Moving toward a Global Age-Friendly Ecosystemubl. J. Am. Geriatr. Soc..

[8] Caniglia G., Luederitz C., von Wirth T., Fazey I., Martín-López B., Hondrila K., König A., von Wehrden H., Schäpke N.A., Laubichler M.D. (2021). A pluralistic and integrated approach to action-oriented knowledge for sustainability. Nat. Sustain..

[9] Chrysikou E. (2018). Why we need new architectural and design paradigms to meet the needs of vulnerable people. Palgrave Commun..

[10] Rowles G.D. (2013). Environmental Gerontology: Making Meaningful Places in Old Age.

[11] Luciano A., Pascale F., Polverino F., Pooley A. (2020). Measuring Age-Friendly Housing: A Framework. Sustainability.

[12] Wang S., Bolling K., Mao W., Reichstadt J., Jeste D., Kim H.C., Nebeker C. (2019). Technology to Support Aging in Place: Older adults’ perspectives. Healthcare.

[13] Sandholdt C.T., Cunningham J., Westendorp R., Kristiansen M. (2020). Towards Inclusive Healthcare Delivery: Potentials and Challenges of Human-Centred Design in Health Innovation Processes to Increase Healthy Aging. Int. J. Environ. Res. Public Health.

[14] Ossokina I.V., Arentze T.A., van Gameren D., van den Heuvel D. (2020). Best living concepts for elderly homeowners: Combining a stated choice experiment with architectural design. J. Hous. Built Environ..

[15] Arup H.A. (2015). Intel, Systematica: Shaping Ageing Citie; 10 European Case Studies. https://ifa.ngo/publication/demographics/shaping-ageing-cities-10-european-case-studies.

[16] AARP-Binette J. (2017). Livability for All: The AARP 2017 Age-Friendly Community Surveys. https://www.aarp.org/research/topics/community/info-2017/aarp-2017-age-friendly-community-surveys.html.

[17] Feng I.M., Chen J.-H., Zhu B.-W., Xiong L. (2018). Assessment of and Improvement Strategies for the Housing of Healthy Elderly: Improving Quality of Life. Sustainability.

[18] Stewart J., Crockett R., Gritton J., Stubbs B., Pascoe A. (2014). Ageing at home? Meeting housing, health and social needs. J. Integr. Care.

[19] Garin N., Olaya B., Miret M., Ayuso-Mateos J.L., Power M., Bucciarelli P., Haro J.M. (2014). Built environment and elderly population health: A comprehensive literature review. Clin. Pract. Epidemiol. Ment. Health.

[20] Wu W., Kaushik I. (2015). Design for Sustainable Aging: Improving Design Communication Through Building Information Modeling and Game Engine Integration. Procedia Eng..

[21] Iwarsson S., Wahl H.W., Nygren C., Oswald F., Sixsmith A., Sixsmith J., Széman Z., Tomsone S. (2007). Importance of the home environment for healthy aging: Conceptual and methodological background of the European ENABLE-AGE Project. Gerontologist.

[22] (ILC-BR) I-BILCB Active Ageing: A Policy Framework in Response to the Longevity Revolution. 2015; p. 116. https://www.matiainstituto.net/en/publicaciones/active-ageing-policy-framework-response-longevity-revolution.

[23] Wahl H.-W., Iwarsson S., Oswald F. (2012). Aging Well and the Environment: Toward an Integrative Model and Research Agenda for the Future. Gerontologist.

[24] Burholt V., Roberts M.S., Musselwhite C.B. (2016). Older People’s External Residential Assessment Tool (OPERAT): A complementary participatory and metric approach to the development of an observational environmental measure. BMC Public Health.

[25] Mulliner E., Riley M., Maliene V. (2020). Older adults’s Preferences for Housing and Environment Characteristics. Sustainability.

[26] MOI (2019). Demographic Statistics. https://www.ris.gov.tw/app/portal/346.

[27] CEPD (2010). The Population Projection in Taiwan from 2010 to 2060. https://pop-proj.ndc.gov.tw/upload/download/Population%20Projections%20for%20the%20Republic%20of%20China%20(Taiwan)-2020~2070.pdf.

[28] NDC (2018). Population Projections for Taiwan: 2018–2065. https://pop-proj.ndc.gov.tw/upload/download/Population%20Projections%20for%20the%20R.O.C%20(Taiwan)%202018%EF%BD%9E2065.pdf.

[29] MOI (2022). Household registration Statistics Data. https://www.ris.gov.tw/app/en/2120.

[30] MOHW Taiwan Health and Welfare Report, Department of Planning 2021. https://www.mohw.gov.tw/dl-77237-0330e4a5-724e-43a4-9ab4-06caec8958bf.html.

[31] Huang W.-H., Lin Y.-J., Lee H.-F. (2019). Impact of Population and Workforce Aging on Economic Growth: Case Study of Taiwan. Sustainability.

[32] Ogawa T., Uchida Y., Nishita Y., Tange C., Sugiura S., Ueda H., Shimokata H. (2019). Hearing-impaired older adults have smaller social networks: A population-based aging study. Arch. Gerontol. Geriatr..

[33] Mosca I., van der Wees P.J., Mot E.S., Wammes J.J.G., Jeurissen P.P. (2017). Sustainability of Long-term Care: Puzzling Tasks Ahead for Policy-Makers. Int. J. Health Policy Manag..

[34] Dorfman J.H., Mandich A.M. (2016). Senior migration: Spatial considerations of amenity and health access drivers*. J. Reg. Sci..

[35] Golant S. Aging in the Right Place; 2015. https://www.researchgate.net/publication/269818962_Aging_in_the_Right_Place.

[36] Wiles J.L., Leibing A., Guberman N., Reeve J., Allen R.E. (2012). The Meaning of “Aging in Place” to Older People. Gerontologist.

[37] Brugger L. (2020). Aging Resiliently: A New Approach to Retirement Housing. https://www.aia.org/articles/149801-aging-resiliently-a-new-approach-to-retirem:56.

[38] CDC (2009). Centers for Disease Control and Prevention, National Center for Chronic Disease Prevention and Health Promotion. Promoting Physical Activity: A Guide for Community Action. https://www.cdc.gov/nccdphp/dnpa/pahand.htm.

[39] WHO (2007). Global Age-Friendly Cities: A Guide. https://apps.who.int/iris/handle/10665/43755.

[40] Rantanen T., Portegijs E., Kokko K., Rantakokko M., Törmäkangas T., Saajanaho M. (2019). Developing an Assessment Method of Active Aging: University of Jyvaskyla Active Aging Scale. J. Aging Health.

[41] Rogelj V., Bogataj D. (2019). Social infrastructure of Silver Economy: Literature review and Research agenda. IFAC-PapersOnLine.

[42] Steels S. (2015). Key characteristics of age-friendly cities and communities: A review. Cities.

[43] Chaudhury H., Oswald F. (2019). Advancing understanding of person-environment interaction in later life: One step further. J. Aging Stud..

[44] Lawton M.P., Nahemow L. (1973). Ecology and the aging process. The Psychology of Adult Development and Aging.

[45] Granbom M., Iwarsson S., Kylberg M., Pettersson C., Slaug B. (2016). A public health perspective to environmental barriers and accessibility problems for senior citizens living in ordinary housing. BMC Public Health.

[46] Prattley J., Buffel T., Marshall A., Nazroo J. (2020). Area effects on the level and development of social exclusion in later life. Soc. Sci. Med..

[47] Buffel T.H., Phillipson C. (2018). Age-Friendly Cities and Communities a Global Perspective.

[48] WHO WHO: Global Strategy and Action Plan on Ageing and Health. 2017; p. 46. https://apps.who.int/iris/handle/10665/329960.

[49] Gammonley D., Kelly A., Purdie R. (2019). Anticipated Engagement in a Village Organization for Aging in Place. J. Soc. Serv. Res..

[50] Lak A., Rashidghalam P., Myint P.K., Baradaran H.R. (2020). Comprehensive 5P framework for active aging using the ecological approach: An iterative systematic review. BMC Public Health.

[51] Ling T.-Y. (2021). Investigating the malleable socioeconomic resilience pathway to urban cohesion: A case of Taipei metropolitan area. Environ. Dev. Sustain..

[52] Sánchez-González D., Rojo-Pérez F., Rodríguez-Rodríguez V., Fernández-Mayoralas G. (2020). Environmental and Psychosocial Interventions in Age-Friendly Communities and Active Ageing: A Systematic Review. Int. J. Environ. Res. Public Health.

[53] Bovaird T., Loeffler E. (2013). The Role of Co-Production for Better Health and Wellbeing: Why We Need to Change. Co-Production of Health and Wellbeing in Scotland.

[54] IDEO (2015). The Field Guide to Human-Centered Design. https://d1r3w4d5z5a88i.cloudfront.net/assets/guide/Field%20Guide%20to%20Human-Centered%20Design_IDEOorg_English-0f60d33bce6b870e7d80f9cc1642c8e7.pdf.

[55] Brown T., Katz B. (2011). Change by Design. J. Prod. Innov. Manag..

[56] Tribess S., Virtuoso Júnior J.S., Oliveira R.J. (2012). Physical activity as a predictor of absence of frailty in the elderly. Rev. Assoc. Med. Bras..

[57] Charmaz K. (2005). Grounded Theory in the 21st Century: Applications for Advancing Social Justice Studies. The Sage Handbook of Qualitative Research.

[58] Skinner M.N., Andrews G.J., Cutchin M.P. (2017). Geographical Gerontology: Perspectives, Concepts, Approaches.

[59] Pankratova N.D., Malafeeva L.Y. (2012). Formalizing the consistency of experts’ judgments in the Delphi method. Cybern. Syst. Anal..

[60] Alabi Tta O.M. (2018). Impact of Information and Communication Technology (Ict) Facilities Deployment on Quantity Surveying Practice in Abuja. http://repository.futminna.edu.ng:8080/jspui/handle/123456789/3127.

[61] Pallant J. (2005). SPSS Survival Manual: A Step by Step Guide to Data Analysis Using SPSS for Windows (Version 12). https://www.taylorfrancis.com/books/mono/10.4324/9781003117452/spss-survival-manual-julie-pallant.

[62] Holt-Lunstad J., Smith T.B., Layton J.B. (2010). Social relationships and mortality risk: A meta-analytic review. PLoS Med..

[63] Bullo V., Bergamin M., Gobbo S., Sieverdes J.C., Zaccaria M., Neunhaeuserer D., Ermolao A. (2015). The effects of Pilates exercise training on physical fitness and wellbeing in the elderly: A systematic review for future exercise prescription. Prev. Med..

[64] Penney L. (2013). The Uncertain Bodies and Spaces of Aging in Place. Anthropol. Aging Q..

[65] Bertelsen P., Kanstrup A.M., Olsson S. (2015). Patient perspectives on patient participation—Results from a workshop with a patient council in a general practice. Driv. Qual. Inform. Fulfilling Promise.

[66] Jayantha W.M., Qian Q.K., Yi C.O. (2018). Applicability of ‘Aging in Place’ in redeveloped public rental housing estates in Hong Kong. Cities.

[67] Crews D.E., Zavotka S. (2006). Aging, disability, and frailty: Implications for universal design. J. Physiol. Anthropol..

[68] Regnier V. (2018). Housing Design for an Increasingly Older Population: Redefining Assisted Living for the Mentally and Physically Frail.

[69] Scheidt R.J. (2019). An interview with author Victor Regnier, FAIA. J. Aging Environ..

[70] Bowes A., Dawson A., Greasley-Adams C., McCabe L. (2016). Design of residential environments for people with dementia and sight loss: A structured literature review. Br. J. Vis. Impair..

[71] Griffiths H. Social Isolation and Loneliness in the UK Report. *Future Cities Catapult* 2016. https://iotuk.org.uk/wp-content/uploads/2017/04/Social-Isolation-and-Loneliness-Landscape-UK.pdf.

[72] Lawton M.P. (1982). Competence, environmental press, and the adaptation of older adults. Aging and the Environment Theoretical Approaches.

